# Impact of Iodinated Contrast on Renal Function and Hemodynamics in Rats with Chronic Hyperglycemia and Chronic Kidney Disease

**DOI:** 10.1155/2016/3019410

**Published:** 2016-02-29

**Authors:** Sheila Marques Fernandes, Daniel Malisani Martins, Cassiane Dezoti da Fonseca, Mirian Watanabe, Maria de Fátima Fernandes Vattimo

**Affiliations:** ^1^Experimentation Laboratory in Animal Model, School of Nursing, University of São Paulo, São Paulo, Brazil; ^2^Department Medical-Surgical Nursing, School of Nursing, University of São Paulo, São Paulo, Brazil

## Abstract

Iodinated contrast (IC) is clinically used in diagnostic and interventional procedures, but its use can result in contrast-induced acute kidney injury (CI-AKI). Chronic kidney disease (CKD) and chronic hyperglycemia (CH) are important predisposing factors to CI-AKI. The aim of this study was to investigate the impact of iodinated contrast on the renal function and hemodynamics in rats with chronic hyperglycemia and chronic kidney disease. A total of 30 rats were divided into six groups; Sham: control of chronic renal disease; Citrate: control of chronic hyperglycemia (CH); Nx5/6: rats with 5/6 nephrectomy; Chronic Hyperglycemia: rats receiving Streptozotocin 65 mg/kg; Nx5/6 + IC: rats Nx5/6 received 6 mL/kg of IC; CH + IC: Chronic hyperglycemia rats receiving 6 mL/kg of IC. Renal function (inulin* clearance*; urinary neutrophil gelatinase-associated lipocalin, NGAL) and hemodynamics (arterial blood pressure; renal blood flow; renal vascular resistance) were evaluated. Iodinated contrast significantly increased urinary NGAL and reduced inulin* clearance*, while the hemodynamics parameters showed changes in arterial blood pressure, renal blood flow, and renal vascular resistance in both CKD and CH groups. The results suggest that the iodinated contrast in risk factors models has important impact on renal function and hemodynamics. NGAL was confirmed to play a role of highlight in diagnosis of CI-AKI.

## 1. Introduction

Contrast-induced acute kidney injury (CI-AKI) is an iatrogenic complication secondary to iodinated contrast (IC) exposure in diagnostic and interventional procedures [1, 2]. It is the third leading cause of hospital-acquired acute kidney injury (AKI) and accounts for about 11% of cases, with adverse effects on prognosis and health care costs [2, 3]. Chronic kidney disease (CKD), chronic hyperglycemia (CH), hypertension, and cardiovascular diseases are important predisposing factors for CI-AKI. The incidence in these individuals may reach 20–50% [4–6].

It has been shown that the administration of IC can result in significant increases in serum creatinine levels and urinary and serum NGAL levels and decrease on glomerular filtration rate (GFR) by reduction of creatinine or inulin* clearance* [7, 8]. The renal vulnerability at CKD is featured by loss of nephron units with reduction on renal function. The renal damage caused by chronic hyperglycemia results in changes in GFR and renal hemodynamics, enhanced tubular transport activity which increases the oxygen consumption, and the generation of reactive oxygen species (ROS) [9]. Therefore, IC exposure in these situations has been associated with important changes in renal function and hemodynamics [10].

The pathogenesis of CI-AKI is unclear; however experimental studies suggest that CI-AKI is due to endothelial dysfunction, inflammation, cellular toxicity, and tubular apoptosis resulting in hemodynamic alterations, hypoxia, and oxidative damage [1, 8]. The use of IC has demonstrated the vasoconstriction effect induced by adenosine and endothelin through a direct receptor-mediated event. Initially, it increases the renal blood flow followed by constriction of renal vasculature [8]. Additionally, the new kidney function markers of CI-AKI have been shown to improve the early detection of renal injury. NGAL has been extensively studied in the field of AKI. In the late phase of AKI, NGAL is believed to play a role as a growth and differentiation factor for restoring tubular epithelial function with the assistance of siderophore-iron complexes [11].

The aim of this study was to investigate the impact of IC on renal function and hemodynamics in rats with chronic hyperglycemia and chronic kidney disease.

## 2. Materials and Methods

### 2.1. Animals

Thirty adult male Wistar rats weighing 280–390 g were used. They were housed in a controlled temperature (25°C/77°F) room with alternating light/dark cycles and were allowed free access to water and rat chow before experimentation.

All procedures in this study are consistent with the ethical principles of animal experimentation adopted by the Brazilian College of Animal Experimentation (COBEA) and were approved by the Ethics Commission on the use of animals, University of Sao Paulo (CEUA-ICBUSP), record of protocol 60, leaves 128 of the book 02.

On the 29th day of the protocol the animals were placed in metabolic cages for the measurement of 24 h urinary volume and collection of a urine sample. After this period, the animals were anesthetized for hemodynamics and renal function studies and blood sample collection through puncture of the abdominal aorta. At the end of the experiment, the animals were sacrificed according to guidelines for animal experimentation.

### 2.2. Streptozotocin-Induced Chronic Hyperglycemia Model

In the first day of the protocol, Streptozotocin (STZ; 65 mg/kg) was dissolved in 0.5 mL citrate buffer (0.1 mol/L; pH 4.5) just before the injection and administrated in the caudal vein. The control animals received only 0.5 mL citrate buffer. Blood glucose levels were measured 48 h after the injection to confirm hyperglycemia (Accu-Chek, Roche; measurement range: 10–600 mg/dL). The animals which consistently showed blood glucose level more than 250 mg/dL were considered hyperglycemic and included in the study.

### 2.3. 5/6-Nephrectomy

In the first day of the protocol, the five-sixths nephrectomy (5/6Nx) was performed under anesthesia with 4% sodium thiopental (30 mg/kg), intraperitoneal (i.p.). After laparotomy, the removal of the right kidney and ligation of 2 branches of the left renal artery were performed, and infarction of two-thirds of the left kidney was achieved.

### 2.4. Iodinated Contrast Administration

The animals received 6 mL/kg of IC (meglumine ioxithalamate and sodium) i.p., single dose on the 28th day of both experimental protocols.

### 2.5. Groups

The groups are as follows:

- Sham: control of chronic kidney disease, rats subject only to laparotomy.
- Citrate: control of chronic hyperglycemia model, rats that received 0.5 mL of citrate buffer, intravenous (i.v.).
- Nx5/6: rats subject to nephrectomy of the right kidney and ligation of 2 branches of the left renal artery and received 6 mL/kg/body weight of single saline i.p., injection on 28th day.
- Chronic Hyperglycemia (CH): rats receiving 65 mg/kg of Streptozotocin dissolved in 0.5 mL citrate buffer i.v. and received 6 mL/kg of single saline injection i.p. on 28th day.
- Nx5/6 + IC: rats subject to Nx5/6 and received 6 mL/kg of IC, single injection i.p. on 28th day.
- CH + IC: rats receiving 65 mg/kg Streptozotocin dissolved in 0.5 mL citrate buffer i.v. and received 6 mL/kg of IC i.p. on 28th day.

### 2.6. Experimental Protocol

The rats were anesthetized with sodium thiopental (50 mg/kg i.p.). Tracheostomy was performed and the rats maintained breathing spontaneously. To measure BP and allow blood sampling a PE-60 catheter was inserted into the left carotid artery. BP was assessed using Biopac Systems MP150 (Santa Barbara, CA). Right jugular vein was cannulated with PE-60 for the administration of inulin and fluids. A small abdominal incision was made and the urinary bladder was cannulated with PE-240 catheter, to collect urine samples.

After the surgical procedure, inulin was injected as a loading dose (100 mg/kg), followed by a continuous infusion of 0.04 mL/min. After a 30 min equilibration period, three urine collections and two blood samples were then obtained at the beginning and at the end. The plasm and urine inulin were measured using the anthrone method [12].

At the end of the experiment, a midline incision was performed; the left renal pedicle was carefully dissected, and the renal artery was isolated. Total RBF was monitored by a perivascular ultrasonic flowmeter (T402; Transonic Systems, Bethesda, MD). RVR was calculated by dividing the BP by total RBF. Finally, the distal abdominal aorta was punctured for terminal blood collection.

### 2.7. Renal Function and Hemodynamics

Forty-eight hours after contrast infusion, the renal function was evaluated based on inulin* clearance* (GFR, mL · min^−1^ · 100 g  body  wt^−1^) and urinary NGAL (Rat-NGAL ELISA kit, BioVendor, research and diagnostic products). Also, arterial blood pressure (BP, mmHg) and total renal blood flow (RBF, mL/min) were assessed and renal vascular resistance (RVR, mmHg · mL^−1^ · min^−1^) was calculated.

### 2.8. Statistical Analysis

One-factor analysis of variance (ANOVA) with confidence intervals for the mean and pairwise comparisons was used. Overlapping intervals indicated no difference between treatments, which was subsequently confirmed by the Tukey test. The results are reported as the mean ± SD. Statistical analysis was made with GraphPad Prism (version 3.0). Statistical significance was also attained with values *p* < 0.05.

## 3. Results

### 3.1. Effects of Iodinated Contrast on Kidney Weight/Body Weight Ratio

Sham and Citrate groups were considered control groups for kidney weight/body weight ratio. As shown in Table 1, CH and CKD rats had significantly increased this ratio (Nx5/6: 0.52 ± 0.03 versus Sham: 0.32 ± 0.04; CH + IC: 0.58 ± 0.13, CH: 0.58 ± 0.11 versus Citrate: 0.34 ± 0.05).

### 3.2. Effects of Iodinated Contrast on Renal Function

Chronic hyperglycemic groups had urinary flow significantly increased compared with the citrate group (CH + IC: 0.042 ± 0.01, CH: 0.032 ± 0.01 versus Citrate: 0.016 ± 0.03) (Table 2). CH and Nx5/6 groups had impairment of GFR as demonstrated for decreased inulin* clearance* (Nx5/6: 0.25 ± 0.08 versus Sham: 0.68 ± 0.05; CH: 0.43 ± 0.03 versus Citrate: 0.74 ± 0.30). After IC use, a significant decrease was observed in GFR in CH + IC and Nx5/6 + IC groups (Nx5/6: 0.25 ± 0.08 versus Nx5/6 + IC: 0.09 ± 0.03; CH: 0.43 ± 0.03 versus CH + IC: 0.17 ± 0.03).

The values of renal function parameters obtained for urinary NGAL were significantly higher in IC groups than in control groups (Nx5/6 + IC: 153.49 ± 70.06 versus Sham: 47.97 ± 19.19; CH + IC: 138.14 ± 74.80 versus Citrate: 45.62 ± 0.95).

### 3.3. Effects of Iodinated Contrast on Renal Hemodynamic

Hemodynamic analysis (Table 3) exhibited that Nx5/6 and Nx5/6 + IC had a higher BP than Sham (Nx5/6: 141.40 ± 11.30, Nx5/6 + IC: 120.20 ± 1.79 versus Sham: 88.75 ± 5.20). Significant reduction of RBF in CH and Nx5/6 groups was observed when compared with citrate and Sham groups (*p* < 0.05). After IC exposure, the groups showed significant increase of RVR (CH + IC: 44.78 ± 7.52 versus CH: 20.85 ± 0.32, Citrate: 18.02 ± 2.29; Nx5/6 + IC: 37.82 ± 10.16 versus Nx + 5/6 : 19.73 ± 4.62, Sham: 11.15 ± 1.62).

## 4. Discussion

In our study, IC exposure in CKD and CH models demonstrated significant changes on renal function and hemodynamics. These effects confirm the pathogenic mechanisms of CI-AKI in risk factors models and can lead to preventive strategies that reduce the chances of kidney damages. NGAL was showed to be an important biomarker at the detection of CI-AKI.

Evidence has shown that the use of IC is associated with a nephrotoxic risk [13]. The presence of CKD and CH increases the risks of IC; also, the morphological alterations on nephrons contributed to direct IC toxicity [5, 6, 9]. Previous reports have shown that renal hypertrophy is a classical feature of Nx5/6 nephrectomy and diabetes models [14, 15]. In the present study, the elevation on KW/BW ratio in the both chronic models could be associated with the hypertrophy, which is due to glomerular basement membrane (GBM) thickening and expansion of the mesangial matrix. Chronic hyperglycemia and Nx5/6 nephrectomy models are characterized by an inflammatory response and accumulation of extracellular matrix (ECM) proteins such as fibronectin, collagen, and laminin [15].

Renal function was evaluated by urinary NGAL and the GFR by inulin* clearance*. Several studies have shown that NGAL presents greater sensitivity for the early detection of AKI [7, 11]. A short period of ischemia (30 min) is sufficient to produce important renal damage in either morphology or function with significant elevations in urinary and serum NGAL [16]. Our results highlighted important increase in urinary NGAL in the CKD and CH groups that were exposed to IC. First, the reduction of inulin* clearance* was almost 50% in CKD and CH animals confirming the renal dysfunction on chronic models. Second, the use of IC on chronic models reduced more than 30% of renal function, resulting in severe CI-AKI. Fonseca et al. [17] also reported a significant reduction in creatinine* clearance* in diabetic rats that were exposed to IC. Third, CI-AKI is associated with a renal insufficiency and hypertension secondary to renin-dependent hypertension in the CKD model and in the chronic hyperglycemia model it results in increased renal plasmatic flow, glomerular hyperfiltration, and hyperosmolar urine [18–20].

Our results demonstrated that use of IC decreased RBF and, consequently, increased RVR in both 5/6 nephrectomy and chronic hyperglycemia rats. The use of IC resulted in severe impairment in renal hemodynamics by constriction of renal vasculature that reduces RBF and induces the development of CI-AKI. The CI-AKI has been associated with the changes in renal hemodynamics through increase of renal vasoconstrictors activity, as vasopressin, angiotensin II, dopamine-1, endothelin, and adenosine, and decreased activity of renal vasodilators, such as nitric oxide and prostaglandins [1, 21–23]. The decreased RBF may also be attributed to increased viscosity or high osmolality of contrast media and increased erythrocyte aggregation, which results in diminished oxygen delivery. The high oxygen consumption results in generation of ROS and cellular apoptosis [1, 8, 24, 25].

CI-AKI is not common in patients with normal renal function; rather, it occurs frequently in patients with CKD and CH. Thus, rats with 5/6 nephrectomy develop hypertension, proteinuria, while the chronic hyperglycemia is associated with intrarenal vasoconstriction that compromises the RBF, resulting in some mechanisms that may be responsible for decrease of GFR [19, 23].

In conclusion, our data confirms that the presence of risk factors for renal dysfunction, as CKD and CH, contributes to enhancing the vulnerability to IC nephrotoxicity, once the renal impairment was significantly higher in animals twice insulted: CKD and CH treated with IC. Additionally, NGAL played an important role in signaling CI-AKI in these models. However, as in this study we could not account for interaction between risk factors, future investigations involving clinical practice data to experimental models, in a translational view, are needed to confirm the mechanisms involved in the CI-AKI.

## Figures and Tables

**Table 1:** Body weight and kidney weight ratio (BW/KW).

Group	*n*	Weight (grams)	BW/KW
Sham	5	404.40 ± 20.90	0.32 ± 0.04
Citrate	5	378.86 ± 49.30	0.34 ± 0.05
Nx5/6	5	340.80 ± 18.30	0.52 ± 0.03^a^
CH	5	277.43 ± 35.72	0.58 ± 0.11^b^
Nx5/6 + IC	5	322.40 ± 14.40^a^	0.43 ± 0.06
CH + IC	5	274.38 ± 36.25	0.58 ± 0.13^b^

^a^ *p* < 0.05 versus Sham.

^b^ *p* < 0.05 versus Citrate.

**Table 2:** Renal function.

Group	*n*	Urinary flow (mL/mim)	Inulin* clearance*/100 g (mL/min/100 g)	Urinary NGAL (pg/mL)
Sham	5	0.013 ± 0.003	0.68 ± 0.05	47.97 ± 19.19
Citrate	5	0.011 ± 0.002	0.74 ± 0.30	45.62 ± 0.95
Nx5/6	5	0.014 ± 0.004	0.25 ± 0.08^b^	76.75 ± 31.33
CH	5	0.032 ± 0.014^a^	0.43 ± 0.03^a^	53.72 ± 21.02
Nx5/6 + IC	5	0.021 ± 0.005	0.09 ± 0.03^bc^	153.49 ± 70.06^b^
CH + IC	5	0.042 ± 0.009^a^	0.17 ± 0.03^ad^	138.14 ± 74.80^a^

^a^ *p* < 0.05 versus Citrate.

^b^ *p* < 0.001 versus Sham.

^c^ *p* < 0.001 versus Nx5/6.

^d^ *p* < 0.001 versus CH.

**Table 3:** Renal hemodynamics.

Groups	*n*	Cardiac frequency (bpm)	Blood pressure (mmHg)	Renal blood flow (mL/min)	Renal vascular resistance, (mmHg·mL^−1^·min^−1^)
Sham	5	464.00 ± 57.40	88.75 ± 5.20	9.28 ± 1.73	11.15 ± 1.62
Citrate	5	417.83 ± 44.59	100.71 ± 6.57	8.38 ± 0.84	18.02 ± 2.29
Nx + 5/6	5	503.80 ± 57.00	141.40 ± 11.30^a^	6.74 ± 1.73^a^	19.73 ± 4.62
CH	5	399.25 ± 63.60	98.93 ± 8.63	4.60 ± 0.28^b^	20.85 ± 0.32
Nx + 5/6 + IC	5	539.20 ± 60.20	120.20 ± 1.79^a^	3.64 ± 1.38^ac^	37.82 ± 10.16^ac^
CH + IC	5	385.37 ± 115.32	97.65 ± 7.22	2.16 ± 0.60^bd^	44.78 ± 7.52^bd^

^a^ *p* < 0.05 versus Sham.

^b^ *p* < 0.05 versus Citrate.

^c^ *p* < 0.05 versus Nx + 5/6.

^d^ *p* < 0.05 versus CH.
